# Development and validation of an interactive educational technology on spotted fever*

**DOI:** 10.1590/1518-8345.3678.3375

**Published:** 2020-09-30

**Authors:** Gabriela Rodrigues Bragagnollo, Rosangela Andrade Aukar de Camargo, Marcela das Neves Guimarães, Tâmyssa Simões dos Santos, Estela Leite Meirelles Monteiro, Beatriz Rossetti Ferreira

**Affiliations:** 1 Universidade de São Paulo, Escola de Enfermagem de Ribeirão Preto, PAHO/WHO Collaborating Centre for Nursing Research Development, Ribeirão Preto, SP, Brazil.; 2 Scholarship holder at the Fundação de Amparo à Pesquisa do Estado de São Paulo (FAPESP), Brazil.; 3 Centro Universitário Maurício de Nassau, Curso de Enfermagem, Maceió, AL, Brazil.; 4 Universidade Federal de Pernambuco, Recife, PE, Brazil.

**Keywords:** Rocky Mountain Spotted Fever, Health Education, Educational Technology, Association Learning, Teaching Materials, Epidemiology, Febre Maculosa, Educação em Saúde, Tecnologia Educacional, Aprendizagem por Associação, Materiais de Ensino, Epidemiologia, Fiebre Maculosa de las Montañas Rocosas, Educación en Salud, Tecnología Educacional, Aprendizaje por Asociación, Materiales de Enseñanza, Epidemiología

## Abstract

**Objective:**

to develop and validate an interactive educational technology on spotted fever, to offer an innovative teaching method.

**Method:**

a methodological study that covered the following stages: analysis and diagnosis; instructional planning, didactic design, review, and validation and production of technology.

**Results:**

the analysis and diagnosis were obtained from experiences in education and health activities for spotted fever. In the instructional planning, it was defined that the technology would be presented in the form of an Interactive Laboratory, with learning stations. The production of the Laboratory was carried out by a multidisciplinary team made up of a carpenter, an electrician, and a plastic artist, among others. The review and validation process was subdivided into two stages: appearance and content validation by professionals in the fields of biology, and education and semantic validation by students of the Nursing and Pedagogy courses. The results of the appearance and content validation showed a content validity index over 0.8 for the vast majority of the variables. In the semantic validation, the Laboratory was evaluated positively by the students.

**Conclusion:**

the trajectory followed for the construction of the Interactive Laboratory on spotted fever gave academic and scientific support to the product, offering an innovative educational resource with pedagogical potential that values significant learning.

## Abstract (pt)

**Objetivo:**

desenvolver e validar uma tecnologia educacional interativa sobre febre maculosa, para oferecer um método inovador de ensino.

**Método:**

estudo metodológico desenvolvido nas seguintes etapas: análise e diagnóstico; planejamento instrucional, desenho didático, revisão e validação e produção da tecnologia.

**Resultados:**

a análise e diagnóstico foram obtidos a partir de experiências em atividades de educação e saúde para febre maculosa. No planejamento instrucional, definiu-se que a tecnologia seria apresentada em forma de Laboratório Interativo, com estações de aprendizagem. A produção do Laboratório foi realizada por uma equipe multidisciplinar constituída por marceneiro, eletricista, artista plástico, dentre outros. O processo de revisão e validação foi subdividido em duas etapas: validação de aparência e conteúdo por profissionais das áreas de biologia e educação e validação semântica por alunos do curso de enfermagem e pedagogia. Os resultados da validação de aparência e conteúdo mostraram um índice de validade de conteúdo superior a 0,8 para a grande maioria das variáveis. Na validação semântica, o Laboratório foi avaliado de forma positiva pelos alunos.

**Conclusão:**

a trajetória percorrida para a construção do Laboratório Interativo sobre febre maculosa conferiu sustentação acadêmica e científica ao produto construído, oferecendo um recurso educativo inovador com potencial pedagógico que valoriza a aprendizagem significativa.

## Abstract (es)

**Objetivo:**

desarrollar y validar una tecnología educativa interactiva sobre fiebre maculosa, para ofrecer un método innovador de enseñanza.

**Método:**

estudio metodológico que recorrió las siguientes etapas: análisis y diagnóstico; planificación instructiva, diseño didáctico, revisión y validación y producción de la tecnología.

**Resultados:**

el análisis y el diagnóstico se obtuvieron a partir de experiencias en actividades de educación y salud para fiebre maculosa. En la planificación instructiva, se definió que la tecnología se presentaría en forma de Laboratorio Interactivo, con estaciones de aprendizaje. La producción del Laboratorio fue realizada por un equipo multidisciplinario constituido por un carpintero, un electricista y un artista plástico, entre otros. El proceso de revisión y validación se subdividió en dos etapas: validación de aspecto y contenido por profesionales de las áreas de biología y educación, y validación semántica por alumnos de las carreras de Enfermería y Pedagogía. Los resultados de la validación de aspecto y contenido mostraron un índice de validez de contenido superior a 0,8 para la gran mayoría de las variables. En la validación semántica, el Laboratorio fue evaluado de manera positiva por los alumnos

**Conclusión:**

la trayectoria recorrida para la construcción del Laboratorio Interactivo sobre fiebre maculosa dio sustentación académica y científica al producto construido, ofreciendo un recurso educativo innovador con potencial pedagógico que valora el aprendizaje significativo.

## Introduction

Spotted Fever (SF) is an emerging zoonosis with high lethality, which has presented itself as a new challenge for public health, since its incidence and prevalence have increased in a worrying way in the last 10 years in Brazil and worldwide. This increase was possibly due to the disease having urbanized, and being no longer limited to rural and forest regions^(^ 1 ^-^ 5 ^)^.

Brazilian SF is an infectious disease caused by the *Rickettsia rickettsii* bacterium, transmitted to man through the bite of the infected *Amblyomma spp* tick. The tick phases that most affect humans are larvae and nymphs, popularly known as micuins and red, which are difficult to visualize and perceive^(^ 6 ^)^. Capybaras have notoriety in the epidemiological chain of the disease, as they are the main reservoirs of the bacteria, in addition to being hosts for SF transmitting ticks^(^ 7 ^)^. The occupation of areas with riparian forests by man brought him closer to the capybaras, which created a favorable environment for the infestation of man by the tick and transmission of the disease^(^ 2 ^)^.

From 2007 to 2017, in Brazil 1,572 cases of SF were confirmed, of which 534 died, adding up to a lethality rate of approximately 34%. The southeastern region of the country has the largest number of cases (980), of which 795 were reported in the State of São Paulo, with 424 deaths^(^ 8 ^)^. In 2018 the state of São Paulo added an alarming number of 103 cases, of which 49 died^(^ 9 ^)^.

As already described, SF is not a disease restricted to Brazil, including other countries in Latin America, such as Colombia, Argentina, and Mexico, which have also registered an increase in the number of cases of the disease. In Argentina, the lethality rate is even higher than in Brazil, being between 40% of the diagnosed cases^(^ 10 ^)^. In Colombia, a study found that, in 2015, the cumulative incidence of infection for the disease reached 6.23%, but this incidence may be even higher due to SF being considered a neglected disease, sometimes underreported when confused with other febrile illnesses^(^ 11 ^)^. A serious mistake, considering that, if SF is not diagnosed and treated in a timely manner, it evolves to death in a few days^(^ 11 ^-^ 13 ^)^. In Mexico, the significant increase in the incidence of SF in 2015 led the country to declare an epidemiological emergency to contain the cases^(^ 14 ^)^.

In the United States, SF is also a public health problem, and its incidence went from 1.7 cases *per* 1 million people in 2000 to 13.2 cases *per* million in 2016^(^ 15 ^)^. In the 2004-2016 period, 650,000 cases of vector-borne diseases were reported, of which 75% were caused by ticks^(^ 16 ^)^.

In order to reduce the problem, the Ministry of Health in Brazil adopted a strategy to identify environments with potential risk for the presence of SF and offered technical training to professionals in the Unified Health System network in these regions^(^ 17 ^)^. To this end, it implemented instructional materials in the format of video lessons on actions of epidemiological and environmental surveillance^(^ 18 ^)^. Unfortunately, these actions were not satisfactory, since the prevalence of the disease is still frequent, which stimulates the development of new actions for preventive education of the population. In fact, one of the gaps observed on the topic in the bibliographic review was the absence of educational materials on the theme, as well as educational interventions in health.

On an ongoing basis, researchers have revealed that health education cannot be limited to just activities that address the transmission of information, since the process of learning demands the construction of strategies in which the didactic and pedagogical aspects meet the cultural dimensions, psychosocial, economic, and political aspects of a given community^(^ 19 ^-^ 20 ^)^. In this sense, when planning health education actions, the researcher needs to recognize the context to detect the factors that may contribute or hinder its development, with the transmission of information being only part of the process^(^ 21 ^)^. The population needs to recognize the need for actions so that they are incorporated into their daily lives, in order to improve the public health scenario in Brazil^(^ 22 ^)^.

In the planning of health education projects, the construction of educational technologies (ETs) that take into account the aforementioned aspects is crucial for the teaching-learning process^(^ 23 ^)^. An ET consists of a systematic set of scientific knowledge that allows planning and execution in order to control, monitor, and evaluate the educational process, so as to interrelate with knowledge and autonomy^(^ 24 ^)^, allowing the individual to live new experiences^(^ 25 ^)^.

It is worth mentioning that the construction of an ET requires the preparation of the educator, in order to understand the elements that make up the teaching-learning process, in addition to genuine respect for the protagonism of the student, arousing curiosity to increase their awareness of the world^(^ 24 ^,^ 26 ^)^. In this perspective, this study was based on Ausubel’s theory of meaningful learning, which values students’ prior knowledge in order to build and reconstruct knowledge based on pleasant and effective learning^(^ 27 ^-^ 28 ^)^.

In this way, a learning environment, based on this theory, seeks to create diverse learning situations with encouragement to interactive learning. For this, it is necessary that the didactic material is potentially significant, to actually involve the student in the understanding of important concepts. In this process, it is crucial to value the students’ previous knowledge, based on their critical and creative thinking, which provides the integration of existing knowledge with the new one^(^ 27 ^-^ 28 ^)^.

The current study aimed to develop and validate an interactive ET on SF to offer an innovative teaching method.

## Method

This was a methodological study^(^ 29 ^)^, based on the stages proposed by Abreu^(^ 30 ^)^: planning (analysis and diagnosis and instructional planning), production (didactic design and review and validation), implementation and evaluation (evaluation). Here, we will present the planning and production stages. The implementation and evaluation of the ET was planned to be carried out in the future.

The analysis and diagnostic evaluation of this study was based on results obtained in a previous extension project, entitled “Spotted Fever: What do I have to do with this?”, offered at USP Campus Units in Ribeirão Preto: Center for Physical Education, Sports and Recreation; Ribeirão Preto Nursing School (*Escola de Enfermagem Ribeirão Preto* - EERP) and at the University Restaurant^(^ 31 ^)^.

The results apprehended in the extension project were expanded by a literature review, with the following guiding question: What knowledge is needed to encourage prophylaxis for SF?

The electronic databases selected for the searches were the US National Library of Medicine/Medical Literature Analysis and Retrieval System Online (PubMed/MEDLINE), Web of Science, Science Direct, Cumulative Index to Nursing and Allied Health Literature (CINAHL), Scientific Electronic Library Online (SciELO), Latin American and Caribbean Literature in Health Sciences (*Literatura Latino-Americana e do Caribe em Ciências da* Saúde, LILACS), and the Nursing Database (*Base de Dados em* Enfermagem, BDENF), the latter two being indexed in the Virtual Health Library (VHL).

To conduct the search, we used the descriptors indexed in the Health Sciences Descriptors (*Descritores em Ciências da* Saúde, DeCS), in Portuguese and Spanish, and descriptors indexed in the Medical Subject Headings (MeSH) for the English language. It is noteworthy that, in PubMed/MEDLINE, Science Direct, Web of Science, and CINAHL we obtained results only with descriptors in English.

The descriptors “Rocky Mountain Spotted Fever”, “Health promotion”, “Prevention”, “Health education”, “Lyme Disease”, “*Fiebre Maculosa de las montañas rocosas*”, “*Promoción de la salud*”, “*Prevención*”, “*Educación en salud*”, “*Enfermedad de Lyme*”, “*Febre maculosa das montanhas rochosas*”, “*Prevenção*”, “*Promoção da saúde*”, “*Educação em saúde*”, and “*Doença de Lyme*” were crossed by the boolean operators “AND” and “AND NOT” in different ways for the maximum production related to the theme.

To compose the list of articles, studies were selected that presented at least one descriptor related to SF and that met the following filter criteria: available in full, database, language, year of publication (2006-2016), and type of document (scientific articles only).

13 searches were carried out, of which 7 (seven) were conducted with combined descriptors and 6 (six) with a single descriptor, with a total of 26 articles selected. The consultation of the literature allowed updating and constructing knowledge on the theme and required reflection on the selection of content to give credibility to the information that would be included in order to fill the knowledge gap of the participants, with an appreciation of the advancement of science on the theme of SF.

The information obtained from the review supported instructional planning, in which the objectives, method, strategies, and specifications of the resources necessary to build the ET were detailed, as well as the content and its sequence^(^ 32 ^)^. The review also contributed to the development of the didactic design of ET on SF, when it was defined that it would be presented in the form of an interactive and self-explanatory laboratory, using learning stations. Thus, the activity was set up to allow the participant to learn in an autonomous, dynamic and playful way, sharpening the use of the senses when exploring and experiencing the educational environments of each station^(^ 33 ^-^ 35 ^)^.

The writing of the script provided a look at the final version of the ET, based on the didactic design^(^ 36 ^)^. A joiner, an electrician, an artist, a tailor, and a draftsman were the professionals responsible for the production of the ET, which took 9 (nine) months to be built. Subsequently, the content, strategies, and proposed activities that constituted the ET were analyzed and evaluated^(^ 30 ^)^ using a specific model^(^ 37 ^)^ to validate its appearance and content. For the semantic validation, the model suggested by the DISABKIDS^®^ group was used, which has been recognized by the scientific community^(^ 38 ^)^.

Professionals in the fields of biology and education of any age and gender, in an intentional non-probabilistic selection, made up a committee of 9 (nine) judges who carried out the validation of the ET’s appearance and content. Thus, we comply with Pasquali’s recommendation^(^ 37 ^)^ with an odd number of specialists to obtain a consistent measurement. After consulting the curriculum vitae on the Lattes Platform, those with more than 5 (five) years of training and who worked in the previously mentioned areas during the information collection period were selected^(^ 39 ^)^.

For the evaluation of the ET, we elaborated an instrument based on another study related to ETs^(^ 40 ^)^, with adjustments, so that the judges were able to evaluate the objectives, content (general organization, structure, presentation, coherence, and formatting), appearance and language (signs expressing ideas and concepts); as well as the clarity, objectivity, ease and understanding of the ET. The items of this instrument were organized in a 5 (five) point Likert scale format^(^ 37 ^)^, where the judges indicated whether they fully agree (5), partially agree (4), neither agree nor disagree (3), partially disagree (2 ) or strongly disagree (1), in addition to providing a space for them to suggest changes. The participation of the judges took place by e-mail, and the instrument was sent along with a document explaining the content of each learning station, photos of it, as well as a video showing the entire ET.

For semantic validation, 8 (eight) undergraduate students from the Nursing and Pedagogy courses were selected for convenience. The number required to complete this phase, according to the DISABKIDS^®38^ manual, is at least 3 (three) participants for each age group and subset of items in the instrument. In the present study, as the age group was not a criterion for differentiating answers, the students were divided according to the specifics of the courses. The sample was probabilistic obtained by manual draw, in order to maintain homogeneity between groups.

The purpose of semantic validation was to ascertain, through interviews with the subjects that compose the population for which the material is intended, the level of understanding and acceptance of the terms, the relevance of the items, the existence of some difficulty, and the possible need of adaptation^(^ 41 ^)^.

Validation took place in two stages: the first evaluated the general impression on the ET in order to identify whether the contents were clear and consistent, using a general impression form. In the second stage, a specific semantic validation was performed, in which the participants analyzed a subset of items, described below.

In accordance with the guidance of the DISABKIDS^®^ Group^(^ 38 ^)^, the students were subdivided into 2 (two) groups to assess the clarity, relevance, and adequacy of each item. Group A was composed of four (4) students from the first year of the Nursing course, who answered the semantic validation form (multiple choice) specific to subset A, composed of 4 (four) variables. In Group B, four (4) students from the first year of the Pedagogy course participated, who answered the semantic validation form (multiple choice) specific to subset B, also composed of 4 (four) variables. The forms used in this phase were made available by the DISABKIDS^®^ group in Brazil^(^ 42 ^-^ 43 ^)^. The limitation of four (4) variables *per* group is justified to avoid tiredness of the research participants.

Descriptive statistics and the Content Validity Index (CVI) were used to analyze the data of the evaluation stage by the committee of judges. The calculation for each variable was performed by dividing the sum of the agreement of the items marked with grades 4 (four) and 5 (five) by the committee of judges by the total number of items answered^(^ 44 ^)^. For semantic validation, data evaluation was also performed using descriptive statistics, by means of the Statistical Package for the Social Sciences (SPSS), version 17.0^(^ 45 ^)^.

The research herein presented was approved by the Research Ethics Committee of the Ribeirão Preto Nursing School - University of São Paulo - CAAE: 57335516.6.0000.5393, following the recommendations of Resolution 466/2012 of the Ministry of Health/National Health Council^(^ 46 ^)^.

## Results

In the stage of diagnostic analysis of previous knowledge on the theme, 210 people attending the USP Campus of Ribeirão Preto participated, 130 people (62%) were female and 80 (38%) male, aged between 18 and 80 years old. The analysis of the answers obtained showed that the population’s knowledge regarding SF was unsatisfactory, as only 35% of the population knew the disease, which could contribute to the risk of exposure since, in the last few years there has been an increase in the number of capybaras inhabiting the Campus, which greatly contributes to the infestation of vegetation by ticks. The identification of the knowledge gap regarding SF stimulated the production of an ET to sensitize the population about the problem and how to prevent it in that environment.

With the identification of the knowledge gap, a review of the literature was carried out to offer current material, seeking to meet the demands of the target audience, valuing the advancement of science on SF. In general, the selected articles pointed out the importance of adopting preventive and health promotion measures, seeking to encourage moments of health education in order to prevent or reduce the risks of SF. The discussions revolved around epidemiological data, incidence, and risks of the disease in specific groups, and suggested preventive measures and educational interventions to combat SF. With regard to health education strategies on the disease, we can highlight the following: 1) Guidelines for people who live in or visit tick-infested areas; 2) Insertion of instructional programs for the community; 3) Conducting workshops; 4) Encouraging people to look for ticks if they have walked through risky places, with immediate removal of ectoparasites; 5) Use of a questionnaire to assess the knowledge of health professionals about the diagnosis and treatment; 6) Use of clothes with insecticides/repellents and, mainly; 7) Use of repellents and barrier methods.

The review studies were quite diverse and mostly dealt with encouraging preventive behaviors in individuals living in and/or visiting areas at risk, so that they can contribute to an early medical diagnosis, in order to reduce the lethality of the disease.

In conjunction with these results, the ET planning was built, in which the principle of significant learning by discovery and reception^(^ 47 ^-^ 48 ^)^ was adopted as a reference. Regarding the discovery, the ET produced is unfinished so that the apprentices can reorganize the set of information to integrate it with their cognitive structures, transforming the construction and creating the desired final product. At reception, the ET was presented to the apprentices in a finished way, requiring only that they internalize the material, which becomes available to be used in the future, providing the freedom to integrate previous knowledge.

**Figure 1:**
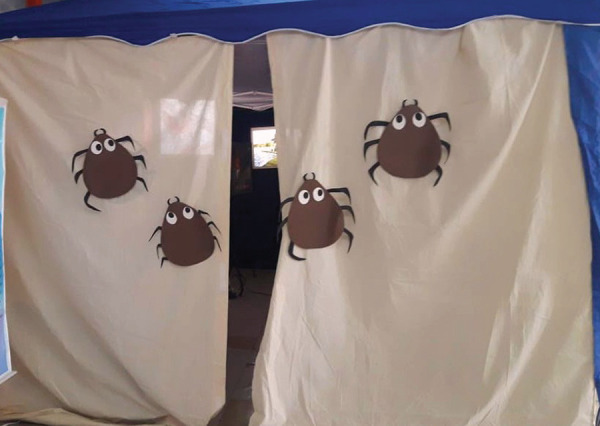
Entrance to the Interactive Laboratory on spotted fever. Image copyright of the author. Ribeirão Preto, SP, Brazil, 2017

In this way, with the plan and script in hand, as well as with the selection of the content, the production of the Interactive Laboratory was carried out. The contents considered relevant were subdivided and organized into 6 (six) learning stations composed of problematic questions to stimulate reflection and curiosity, which questioned the following: What is SF?; How is it transmitted?; Signs and symptoms, Areas of risk, and How to prevent it? The stations will be presented below (Figure 1).

**Figure 2:**
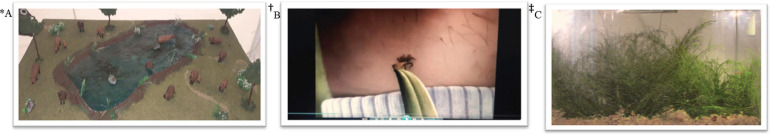
Learning Stations. Ribeirão Preto, SP, Brazil, 2017 *A = Illustrative model of the 1^st^ Station. Image copyright by the author; ^†^B = Video^1^ of the 2^nd^ Station; ^‡^C = Terrarium arranged with live ticks from the 3^rd^ season. Image copyright by the author

1^st^ Station: *Capybara?* What does it represent in the problem? - This station had an illustrative model, representing a risk area, containing a lake, pasture, grass and capybaras. The purpose of this learning station is for the participant to be able to recognize an area at risk for contracting the SF disease, identifying the main animal (capybara) which hosts ticks that may be contaminated with the bacteria that causes SF (Figure 2*A).

2^nd^ Station: *Star Tick or Micuim?* - At this station, the participant watches a video that portrays the moment when a person is crossing a risky area and is bitten by ticks. Then, through an animation, the video shows the process in which the parasite sucks the host’s blood and regurgitates saliva, which contains the bacteria that causes SF, infecting man. Printed and laminated images were also displayed at this station, showing the evolutionary phases of ticks. The purpose of this learning station is for the participant to be able to understand who is the causative and transmitting agent of the disease, in addition to visualizing the dynamics of the infestation (Figure 2 ^†^B).

3^rd^ Station: *Smart housing!* - To bring excitement and greater reality to the activity, this station contained a terrarium, composed of a seedling of grass planted in a pot and live ticks (nymphs). The purpose of this learning station is for the participant to be able to identify live ticks at the tip of the grass, recognizing how ticks are found in the environment (Figure 2 ^‡^C).

**Figure 3:**
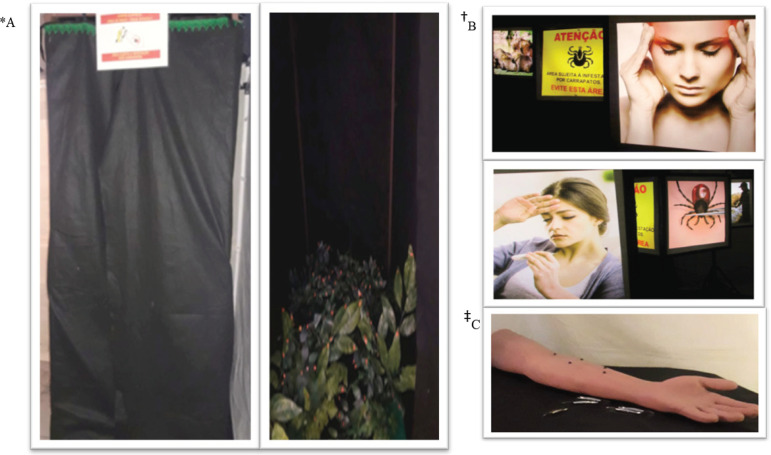
Learning Stations. Ribeirão Preto, SP, Brazil, 2017 *A = 4^th^ station’s sensory booth. Image copyright by the author; ^†^B = 5^th^ station illuminated cubes^2^; ^‡^C = Silicone arm with artificial ticks from the 6^th^ station. Image copyright of the author.

4^th^ Station: *Risk booth, stay tuned!* - The “Senses Booth” was a resource designed for the participant to have the feeling of being in an area at risk for SF infection. Inside this booth, artificial grass was placed on the floor, in which Styrofoam ticks painted with fluorescent paint were adhered. When the participants entered the booth, an automatic movement control switched on a black light which induced flowering in the ticks. The participants also feel the grass brush their legs, which stimulates the senses. The purpose of this learning station is to offer an experience to the participants, simulating the entry into an area of risk, articulating in a practical way information covered in previous stations (Figure 3*A).

5^th^ Station: *Stay informed, avoid ticks!* - Seeking to show areas at risk, prevention, and signs of initial SF symptoms, this station exhibited high definition images in illuminated cubes made of canvas and wood, where each side of the cube showed a different image. In this station, the existence of a health team on the Campus was informed, which, in addition to notifying human parasitism due to ticks for environmental and epidemiological surveillance, guides and directs affected people for medical follow-up. The purpose of this learning station is for the participant to be able to recognize the signs and symptoms of SF and the types of prevention, in addition to reviewing the knowledge previously presented (Figure 3 ^†^B).

6^th^ Station: *What should I do if I find a tick on me?* - To provide a more realistic learning experience, in this station, the participants had the opportunity to remove artificial ticks attached to a human silicone arm with tweezers, so that they could experience and simulate their removal. Still at this station, printed and laminated images were displayed, demonstrating the correct way to remove ticks. The purpose of this learning station is for the participant to know the correct way to remove ticks from the human body, so that tick fragments are not left inside the skin (Figure 3 ^‡^C).

To disseminate the interactive laboratory to the population, a canvas panel was installed, containing information about the location, date, and time of the activity, as well as a QR code (2D barcode), which can be recognized by cell phones (smartphones), notebooks, and tablets, in order to provide access to a link^(^ 49 ^)^ on the Internet that promoted access to an informative text prepared by our research team, called “Spotted Fever - a disease transmitted by ticks”, also available on the website of the Mayoralty of the USP Campus in Ribeirão Preto (*Prefeitura do Campus USP de Ribeirão Preto*, PUSP - RP)^(^ 50 ^)^.

The interactive laboratory on SF sought to create an innovative environment by presenting six learning stations, with content contextualized in an increasing sequence of deepening. Furthermore, when participating in the ET, the apprentices can review the same topic as many times as they deem necessary, in addition to using the time they consider necessary. For this, the laboratory used potentially significant materials to interact in a non-arbitrary way with the individual’s previous experience, offering options of modalities and strategies, in order to contribute to the teaching-learning process.

Throughout the course of the activity, a supervisor was present, acting as an orientator, stimulator, and evaluator of learning, assisting the participants in the task of formulating and reformulating concepts, activating their previous knowledge, and articulating this knowledge with new information.

The next stage of the study was the review and validation of the ET by specialists (judges) in the field of biology and education (appearance and content validation), as well as by representatives of the target population (semantic validation).

The panel of judges who participated in the appearance and content validation included 6 (six) women (66.75%) and 3 (three) men (33.3%), 7 (seven) of whom studied at a state institution (77.8%) and 2 (two) at a private institution (22.2%). All the judges reported having a Master’s degree, 6 (six) of them also reported having a PhD degree (66.7%) and 6 (six) cited having an internship and/or a specialization (66.7%). Of the 9 (nine) judges, 7 (seven) (77.8%) work at the USP and 2 (two) (22.2%) at another institution.

The judges’ answers regarding the appearance and content validation for each of the items assessed in relation to the educational technology are shown in Table 1.

**Table 1:** Distribution of the judges' answers in the validation of the instrument's appearance and content for each question presented according to the Likert scale. Ribeirão Preto, SP, Brazil, 2017

Questions	J*1	J*2	J*3	J*4	J*5	J*6	J*7	J*8	J*9	CVI^†^
Are the stations organized?	5	5	5	5	5	5	5	5	5	1.00
Are the illustrations clear?	5	5	5	5	5	5	5	5	5	1.00
Do the illustrations help to expand the content?	5	5	5	5	5	5	5	5	5	1.00
Is content presentation coherent?	5	5	5	5	5	5	5	5	5	1.00
Is the instructional sequence appealing and logical?	5	5	5	5	5	5	5	5	5	1.00
Does the activity encourage interaction?	4	5	5	5	5	4	5	5	5	1.00
Is the size of the content adequate?	4	4	5	5	5	4	4	5	5	1.00
Is the writing in a sociable style?	3	5	5	5	5	4	5	3	4	0.78
Is any technical jargon included?	3	5	4	5	5	5	5	4	5	0.89
Is the text vivid and interesting? Is the tone friendly?	3	5	4	5	5	5	5	3	5	0.78
Is the text clear?	4	5	5	5	5	4	5	4	5	1.00
Objectivity	4	5	5	5	5	5	5	5	5	1.00
Scope	4	5	4	5	4	5	5	5	5	1.00
Updating	5	5	4	5	5	5	5	5	5	1.00
Vocabulary	4	5	5	5	5	4	4	5	5	1.00
Clarity of the content	4	5	5	5	5	4	5	5	5	1.00
Content presentation in each station	4	5	4	5	5	4	5	5	5	1.00
Instructional sequence	5	5	4	5	5	5	5	5	5	1.00
Objectivity	5	5	5	5	5	5	5	5	5	1.00
Scope	5	5	4	5	5	5	5	5	5	1.00
Updating	5	5	4	5	5	5	5	5	5	1.00
Vocabulary	4	5	5	5	5	4	5	4	5	1.00
Clarity of the content	5	5	5	5	5	4	5	4	5	1.00

* J = Judge;

† CVI = Content Validity Index

As can be seen, the vast majority of the items had a CVI over 0.8, meaning that there is great agreement between the judges. Only in 2 (two) items, “Is the writing in a sociable style?” and “Is the text vivid and interesting? Is the tone friendly?”, the CVI was below 0.8. Thus, 2 (two) judges indicated that they partially agreed, recording comments and suggestions: *As the activity is not intended only for students in the health area, I suggest “popularizing” the language a little, since in some stations the content is written in a very scientific way which can impair learning for the participant who is from another course* (Judge 1); *In the video that the text is ok, it is more colloquial. However, in the printed version there are scientific terms and minor grammar errors that need to be corrected if it is to be disseminated in any environment* (Judge 2).

In view of the judges’ answers, we consider it pertinent to carry out a review of the content, making it more accessible to the population. For this purpose, the technical terms contained in the activity were removed, namely: cephalea and myalgia, in this way we were able to reduce the use of technical jargon, although this item had a CVI over 0.80.

In the general impression semantic validation stage, all the participants considered the interactive laboratory as “very good” (100%), 87.5% referred to ease of understanding, and 100% considered the stations to be important for SF knowledge. No participant suggested changing and/or adding anything to the learning stations.

In the specific semantic validation, where the content of each learning station was assessed separately, all the participants (100%) in Group A considered the content of the stations clear and coherent. In Group B (students from the Pedagogy course), 25% of the participants had difficulty understanding the content of the 5^th^ learning station, which refers to the signs and symptoms of the disease. This result corroborates the judges’ appreciation in the appearance and content validation, which guided the substitution of the words cephalea for headache and myalgia for muscle pain.

The vast majority of the participants in the semantic validation considered that the content of the learning stations was adequate to encourage health prevention and SF control practices, which indicates that the laboratory must have good acceptance and understanding by the target population, eliminating the need for major changes.

Taken together, the validation stages showed that the interactive laboratory on SF had clear and concise information that meets the needs of the target audience; thus, scientific proof was provided which supported the implementation of the ET.

## Discussion

This research developed and validated an ET called “Interactive Laboratory on Spotted Fever” for the population of the USP Campus of Ribeirão Preto, considered a risk area for contracting the disease. The methodological study began with an assessment of the context where the ET will be applied and a description of the knowledge gaps on the topic, configuring the diagnostic analysis. Later, it endorsed the importance of instructional planning, its didactic design, and validation, in order to meet the learning needs, based on the theory of significant learning^(^ 27 ^)^.

The Ausubel framework was chosen for providing the individual inserted in the teaching-learning process, the development of a new concept based on previous knowledge. Some authors have used this framework for the development of ETs aimed at the adult audience, and this has proved to be appropriate to contribute to changes in knowledge in a significant way^(^ 51 ^-^ 52 ^)^.

When analyzing ETs developed in countries like the United States^(^ 53 ^)^, Spain^(^ 54 ^)^, Colombia^(^ 55 ^)^, Chile^(^ 56 ^)^, Venezuela^(^ 57 ^)^, and Brazil^(^ 24 ^,^ 35 ^,^ 58 ^-^ 60 ^)^, it was possible to understand that the best results are directly associated with the interaction that these resources provide. That is, these studies show that it is essential to consider the users’ prior knowledge and doubts in the elaboration of ETs; to this is added the relationship between the quality of educational materials and the use of defined principles and types of elaboration^(^ 61 ^-^ 63 ^)^.

Accordingly, studies carried out in Brazil^(^ 64 ^-^ 65 ^)^, Venezuela^(^ 66 ^)^, United States^(^ 67 ^)^, and Italy^(^ 68 ^)^ that worked with ETs obeying scientific criteria and seeking to know the target population, adopted strategies with the potential to gather modifying knowledge for the teaching-learning process.

The instructional planning of the current study was built in a systematic way to understand the scenario in which we were inserted, facilitating the choice of the approach and the type of action for the transformation of the practice and, consequently, of the reality, and trying to build a creative, consistent, and innovative design.

A number of methodological studies developed by Nursing reveal that creativity associated with scientific knowledge strengthens and expands health education, offering new ways of thinking, organizing and managing care, which provides an innovative environment for the production of knowledge, enabling the autonomy of the subjects and promoting quality of life^(^ 69 ^-^ 70 ^)^.

However, in order for the ET to fulfill its objective, validation as a scientifically reliable product is essential, and it is also tested for its effectiveness and suitability for its application^(^ 69 ^)^. In fact, carrying out the validation of an educational resource is crucial for researchers and health professionals to be able to trust and evaluate whether it is convenient for a given population^(^ 23 ^)^.

A study that aimed to build and validate an ET in the form of a video for people and families who experience colostomy and cancer highlights the importance of validation by specialists, since an educational material, when well produced and validated, can really contribute to modifying the reality of the subjects for whom it is intended^(^ 69 ^)^.

The use of appearance and content validation by specialists (judges) has been adopted by several researchers in ET assessments. Our work, employing this type of validation, obtained CVI values similar to those found in the literature^(^ 64 ^-^ 65 ^,^ 69 ^,^ 71 ^-^ 74 ^)^.

Interestingly, the target audience (semantic validation) also satisfactorily evaluated the interactive laboratory on SF. In addition, the participants brought valuable contributions and did not go to great lengths to collaborate with the improvement of the technology. This validation pointed out confusing and little understandable passages, enabling them to be adjusted and become compatible with popular understanding. Thus, the agreement of the students in relation to the clarity of the interactive laboratory led to a greater probability that the technology would enable the multiplication of information and contribute more effectively to the training of laypeople on the subject^(^ 75 ^)^.

In fact, receiving suggestions and opinions favored the exchange of ideas based on the participants’ daily experiences. The validated technology is not restricted to promoting knowledge about SF, it awakens new ideas in the learners, instigating their curiosity and provoking contextualized and pleasurable reflections involving their health and that of the community in controlling SF.

Therefore, it is worth noting that the elaboration of an ET is not an elementary task, it requires commitment and involvement of a multiprofessional team, from the initial phases of its project to its distribution to the end user^(^ 71 ^)^.

As for the limitation of this study, we can mention the cost for the development of an ET, since there is a demand for third party service contracts and consumables. Therefore, fundraising must be taken into consideration before starting the contract.

The interactive laboratory produced made the most of human potential in the construction of knowledge, by proposing an integration of different ways of apprehending new knowledge, extrapolating the visual and auditory sensations. In addition, it added experiences that transport the apprentices to a scenario very close to the real one, causing feelings and driving attitudes, in order to awaken behavior changes.

Finally, it should be noted that creativity was one of the main pillars for this study, since the individual’s holistic involvement in the teaching-learning process was valued, using the playful and the imaginary through sensory experiences. Still, the theoretical framework which was selected also contributed to the fact that creativity was the main element in the planning of the ET, although always under methodological rigor.

## Conclusion

This methodological study, which produced and validated an ET for health education on SF, was based on the needs of people who frequent an area at risk for the disease. It is considered that the trajectory followed provides academic and scientific support to the product constructed and also contributes with pedagogical potential as a propositive ET with an interactive approach, an essential requirement to value meaningful learning.

The methodology used proved to be able to support the elaboration of an innovative ET, which can instrumentalize the elaboration of other ETs, as well as sensitize health professionals, educators, and researchers for the production and validation of new ETs, both in this theme and in any another that involves health education actions.

Finally, developing an interactive laboratory on SF based on a methodological study can contribute to the empowerment of the population, favoring changes in attitudes. Thus, this work collaborates with the health promotion policy in face of the epidemiological and environmental surveillance actions for SF identified by the Ministry of Health in Brazil.
